# New Types of Wheat Chromosomal Structural Variations in Derivatives of Wheat-Rye Hybrids

**DOI:** 10.1371/journal.pone.0110282

**Published:** 2014-10-10

**Authors:** Zongxiang Tang, Meng Li, Lei Chen, Yangyang Wang, Zhenglong Ren, Shulan Fu

**Affiliations:** Agronomy College, Sichuan Agricultural University, Wenjiang, Chengdu, Sichuan, China; United States Department of Agriculture, United States of America

## Abstract

**Background:**

Chromosomal rearrangements induced by wheat-rye hybridization is a very well investigated research topic. However, the structural alterations of wheat chromosomes in wheat-rye hybrids are seldom reported.

**Methodology/Principal Findings:**

Octoploid triticale lines were derived from common wheat *Triticum. aestivum* L. ‘Mianyang11’×rye *Secale cereale* L. ‘Kustro’. Some progeny were obtained by the controlled backcrossing of triticale with ‘Mianyang11’ and common wheat *T. aestivum* L. ‘Chuannong27’ followed by self-fertilization. Fluorescence *in situ* hybridization (FISH) and genomic *in situ* hybridization (GISH) using Oligo-pSc119.2-1, Oligo-pTa535-1 and rye genomic DNA as probes were used to analyze the mitotic chromosomes of these progeny. Alterations of wheat chromosomes including 5A, 6A, 1B, 2B, 6B, 7B, 1D, 3D and 7D were observed. 5AL arm carrying intercalary Oligo-pSc119.2-1, Oligo-pTa535-1 or both Oligo-pSc119.2-1 and Oligo-pTa535-1 signals, 6AS, 1BS and 1DL arms containing terminal Oligo-pSc119.2-1 signal, 6BS and 3DS arms without terminal Oligo-pSc119.2-1 signal, 7BS without subtelomeric Oligo-pSc119.2-1 signal and 7DL with intercalary Oligo-pSc119.2-1 signal have been observed. However, these changed wheat chromosomes have not been detected in ‘Mianyang11’ and Chuannong 27. The altered 5A, 6A, 7B and 7D chromosomes in this study have not been reported and represent several new karyotype structures of common wheat chromosomes.

**Conclusions/Significance:**

These rearranged wheat chromosomes in the present study afford some new genetic variations for wheat breeding program and are valuable materials for studying the biological function of tandem repetitive DNA sequences.

## Introduction

Wide hybridization is very important for wheat (*Triticum aestivum* L.) cultivar improvement because it can enrich the cultivated gene pools by incorporating favourable alleles, genes or gene complexes from wild relatives [1]. In the program of using wide hybridization methods to improve wheat cultivars, attention was often directed at alien elite gene that has been introgressed into wheat background, however, the structural variations of wheat chromosomes in the derivatives from wheat×wild relatives is notable. Chromosome rearrangements including deletions, translocations, ring chromosomes, dicentric chromosomes and a paracentric inversion were observed during the production of a substitution of chromosome 6B^s^ from *Triticum speltoides* (Tausch) Gren. ex Richter for chromosome 6B of Chinese Spring wheat (*T. aestivum* L.) [2]. In newly synthesized amphiploids of *Aegilops* and *Triticum*, the reduction or amplification of subtelomeric repeated sequences Spelt1 and Spelt52 have been observed [3]. In synthetic amphiploid derived from *T. aestivum* (accession 252) × *Ae. speltoides* (accession 15-1), drastic physical elimination of tandem DNA repeat pGc1R-1 has been detected [4]. Localized genomic alterations have been discovered in newly synthesized allotetraploid wheats [5]. These previous studies indicate that the structure of wheat chromosomes could be changed in derivatives, which were derived from wide hybridization. Wide hybridization between wheat (*T. aestivum* L.) and rye (*Secale cereale* L.) has been successfully used in wheat breeding programs. Alterations of chromosomal structure of rye chromosomes in triticales, wheat-rye disomic addition lines and wheat-rye substitution lines have been discovered [6]–[11]. Genome rearrangements and chromosome instability in wheat-rye disomic addition lines have also been detected [12]–[13]. Structural variations of wheat chromosomes induced by wheat-rye monosomic addition lines have been reported [14]. These studies mentioned above indicated that both wheat and rye chromosomes could change in derivatives from wheat×rye. Although abundant genetic diversity was stored in wheat-rye hybrids, the structural alterations of wheat chromosomes in wheat-rye hybrids are seldom reported. In fact, more attentions should be paid on the variations of wheat chromosomes in wheat-rye hybrids. In the present study, some new types of wheat chromosomal structural variations in derivatives from wheat × rye were discovered and discussed.

## Results

### GISH and FISH analysis of ‘Mianyang11’ and ‘Chuannong27’

**Figure 1:**
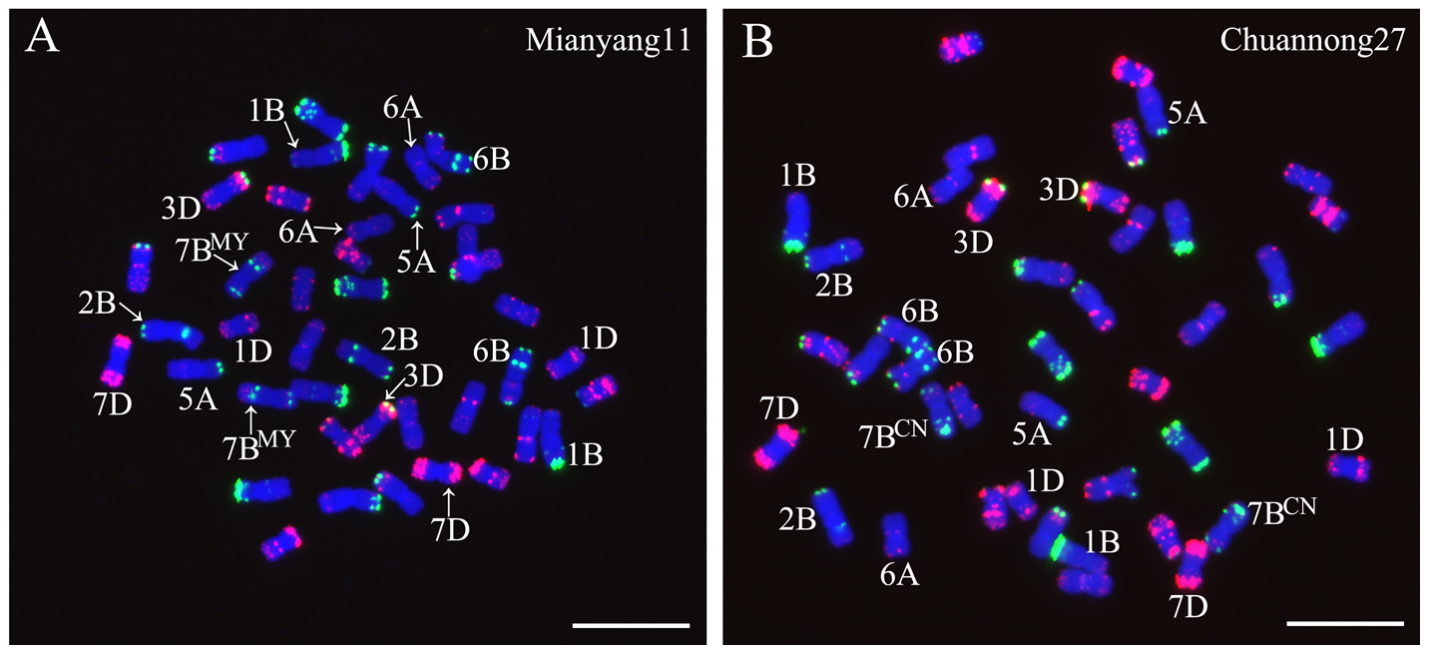
FISH analysis of ‘Mianyang11’ and ‘Chuannong27’ using Oligo-pSc119.2-1 (green) and Oligo-pTa535-1 (red) as probes. **A** FISH pattern of ‘Mianyang11’. **B** FISH pattern of ‘Chuannong27’. The chromosomes, which were involved in structural alterations in this study, are marked. Chromosomes were counterstained with DAPI (blue). Bars = 10 µm.

GISH analysis by using rye genomic DNA as probe indicated that no rye chromatins existed in ‘Mianyang11’ and ‘Chuannong27’ (data not shown). Using Oligo-pTa535-1 and Oligo-pSc119.2-1, enabled the A-, B- and D-genome chromosomes of ‘Mianyang11’ and ‘Chuannong27’ were to be distinguished from each other (Figure 1). Oligo-pTa535-1 hybridized well to wheat D-genome chromosomes and Oligo-pSc119.2-1 mainly hybridized to B-genome chromosomes, as reported by Tang et al. [15]. For ‘Mianyang11’ and ‘Chuannong27’, it can be noted that 5AL arms did not contain strong intercalary signals of Oligo-pTa535-1 and Oligo-pSc119.2-1, terminal Oligo-pSc119.2-1 signals could not be detected on 1BS arms, 6A, 1D and 7D chromosomes did not bear Oligo-pSc119.2-1 signals, and strong signals of Oligo-pSc119.2-1 existed on the terminal regions of 3DS arms, on both the terminal regions of 6B chromosomes and on the subtelomeric regions of 7BS arms (Figure 1). The distal Oligo-pSc119.2-1 signals on 7BL arms of ‘Chuannong27’ were stronger than that on 7BL arms of ‘Mianyang11’ (Figure 1). Therefore, the 7B chromosomes of ‘Mianyang11’ and ‘Chuannong27’ were named 7B^MY^ and 7B^CN^, respectively.

### GISH and FISH analysis of line 1-6-7B-2 and its self-fertilized progeny

**Figure 2:**
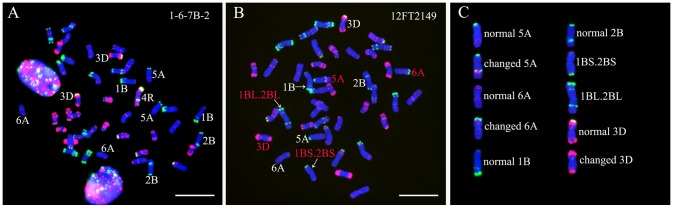
GISH and FISH using rye genomic DNA (red), Oligo-pSc119.2-1 (green) and Oligo-pTa535-1 (red) as probes on lines 1-6-7B-2 and 12FT2149. **A** GISH and FISH pattern of line 1-6-7B-2. **B** Line 12FT2149 contains no rye chromosome and changed wheat chromosomes (marked by red letters). **C** Cut-out chromosomes for the comparison between normal and changed wheat chromosomes. Chromosomes were counterstained with DAPI (blue). Bars = 10 µm.

1-6-7B-2 is a wheat-rye 4R monosomic addition line (Figure 2A). Compared with ‘Mianyang11’, apparent structural alterations of wheat chromosomes in line 1-6-7B-2 have not been detected. A line 12FT2149 without rye chromatin was identified from 30 randomly selected progeny of line 1-6-7B-2. The structural modifications of 5A, 6A and 3D chromosomes in this line have been detected (Figure 2B–C). That is, strong intercalary Oligo-pTa535-1 and Oligo-pSc119.2-1 signals on one 5AL arm were detected, a strong terminal Oligo-pSc119.2-1 signal could be observed on one 6AS arm (Figure 2B–C), and the terminal Oligo-pSc119.2-1 site disappeared from one 3DS arm (Figure 2B–C). In addition, 1BS.2BS and 1BL.2BL translocation chromosomes existed in line 12FT2149 (Figure 2B–C). These two translocation chromosomes were arised from reciprocal translocation between 1B and 2B chromosomes at centromere. No obvious structural variations of wheat chromosomes were observed in the other 29 self-fertilized progeny of line 1-6-7B-2. These results indicate that a single 4R chromosome added into wheat genetic background could induce not only localized structural variation of wheat chromosomes but also reciprocal translocation between wheat chromosomes.

### GISH and FISH analysis of lines 12FT1290 and 12FT1378 and their self-fertilized progeny

**Figure 3:**
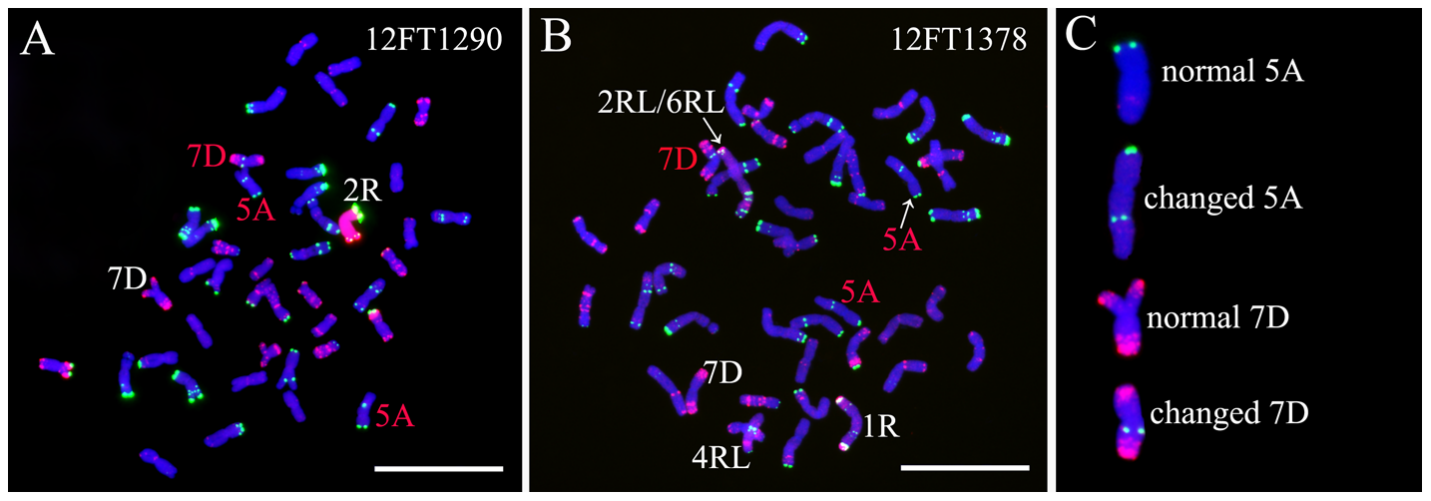
GISH and FISH using rye genomic DNA (red), Oligo-pSc119.2-1 (green) and Oligo-pTa535-1 (red) as probes on lines 12FT1290 and 12FT1378. **A** GISH and FISH pattern of line 12FT1290. **B** GISH and FISH pattern of line 12FT1378. **C** Cut-out chromosomes for the comparison between normal and changed wheat chromosomes. The changed wheat chromosomes are marked by red letters in A and B. Chromosomes were counterstained with DAPI (blue). Bars = 10 µm.

12FT1290 is a wheat-rye 2R monosomic addition line (Figure 3A). Line 12FT1378 contained a 2RL/6RL translocation chromosome, a 4RL arm, and a 1R chromosome (Figure 3B). Compared with their parental wheat ‘Mianyang11’, the structures of 5A and 7D chromosomes in the two lines have changed. That is, strong intercalary Oligo-pSc119.2-1 signals existed on two 5AL arms and one 7DL arm (Figure 3A–C).

**Figure 4:**
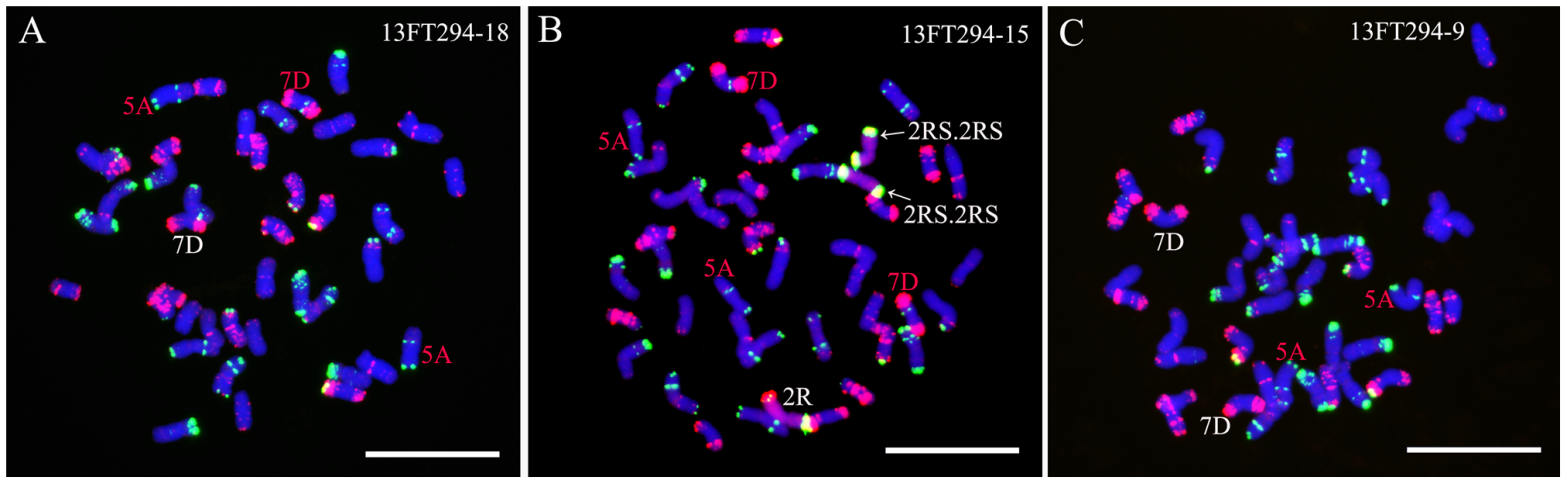
GISH and FISH using rye genomic DNA (red), Oligo-pSc119.2-1 (green) and Oligo-pTa535-1 (red) as probes on progeny of line 12FT1290. **A** GISH and FISH pattern of line 13FT294-18, representing lines with two changed 5A chromosomes and one changed 7D chromosome. **B** GISH and FISH pattern of line 13FT294-15, representing lines with two changed 5A chromosomes and two changed 7D chromosomes. **C** GISH and FISH pattern of line 13FT294-9, representing lines with two changed 5A chromosomes and normal 7D chromosomes. No rye chromosome(s) are detected in lines 13FT294-18 and 13FT294-9. The changed wheat chromosomes are marked by red letters. Chromosomes were counterstained with DAPI (blue). Bars = 10 µm.

Twenty-one seeds (13FT294.1-13FT294.21) were randomly selected from the self-fertilized progeny of 12FT1290 for FISH and GISH analyses. All the 21 seeds contained two changed 5A chromosomes (Figure 4), 12 of the 21 seeds contained one changed 7D chromosomes (Figure 4A), four seeds contained two changed 7D chromosomes (Figure 4B) and five seeds contained unchanged 7D chromosomes (Figure 4C).

**Figure 5:**
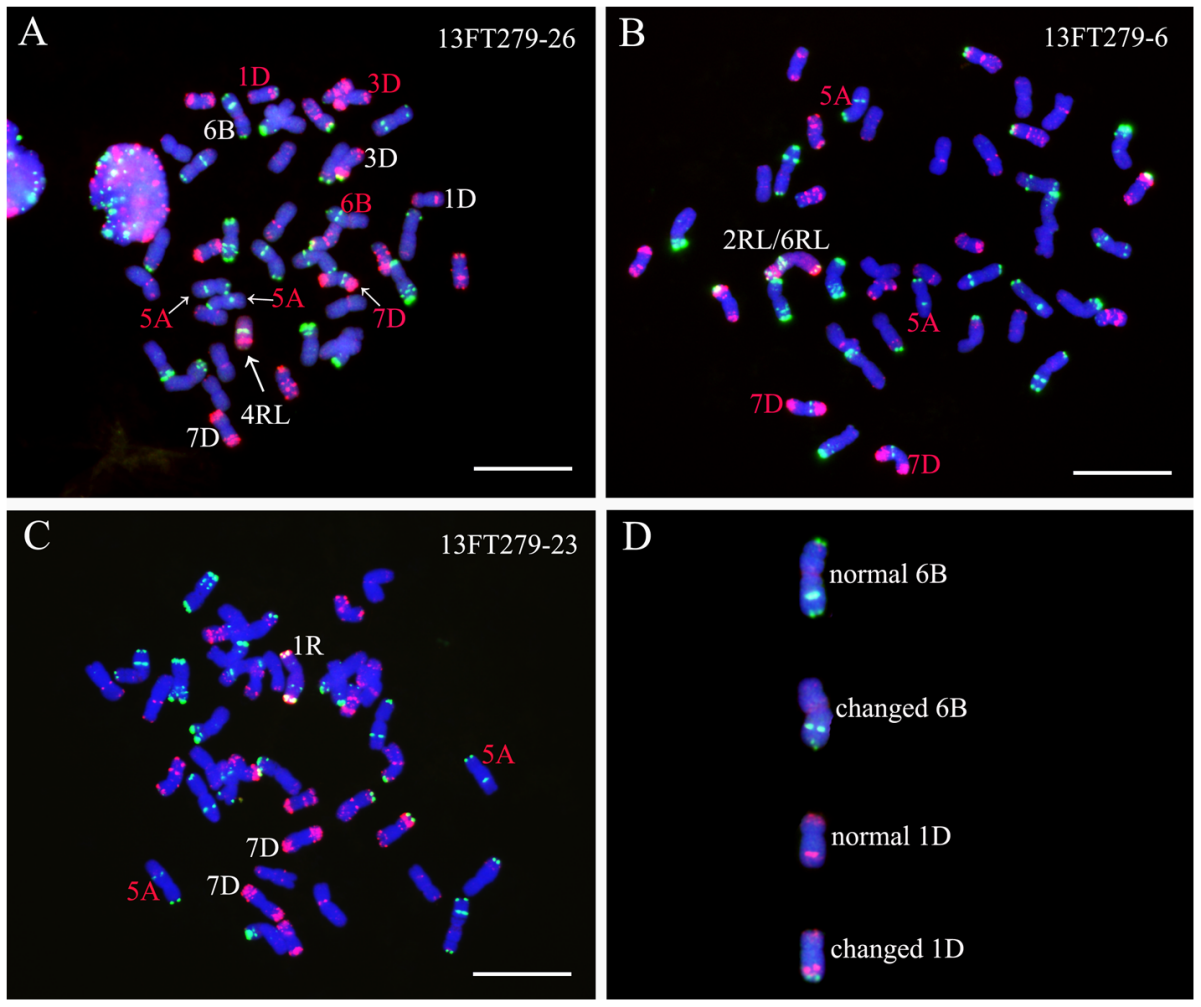
GISH and FISH using rye genomic DNA (red), Oligo-pSc119.2-1 (green) and Oligo-pTa535-1 (red) as probes on progeny of line 12FT1378. **A** GISH and FISH pattern of line 13FT279-26, representing lines with two changed 5A chromosomes and one changed 7D chromosome. **B** GISH and FISH pattern of line 13FT279-6, representing lines with two changed 5A chromosomes and two changed 7D chromosomes. **C** GISH and FISH pattern of line 13FT279-23, representing lines with two changed 5A chromosomes and normal 7D chromosomes. **D** Cut-out chromosomes for the comparison between normal and changed wheat chromosomes. Changed wheat chromosomes are marked by red letters in A, B and C. Chromosomes were counterstained with DAPI (blue). Bars = 10 µm.

Twenty-six seeds (13FT279.1-13FT279.26) were randomly selected from the self-fertilized progeny of 12FT1378 for FISH and GISH analyses. All the 26 seeds contained two changed 5A chromosomes (Figure 5), eight of the 26 seeds contained one changed 7D chromosome (Figure 5A), ten seeds contained two changed 7D chromosome (Figure 5B) and eight seeds contained normal 7D chromosomes (Figure 5C). These results indicate that the changed 5A and 7D chromosomes could be transmitted stably to the offspring. Additionally, in line 13FT279.26, terminal Oligo-pSc119.2-1 signals appeared on a 1DL arm and disappeared from a 3DS arm and a 6BS arm (Figure 5A, D).

### GISH and FISH analysis of lines 12FT1844 and 12FT1997 and their self-fertilized progeny

**Figure 6:**
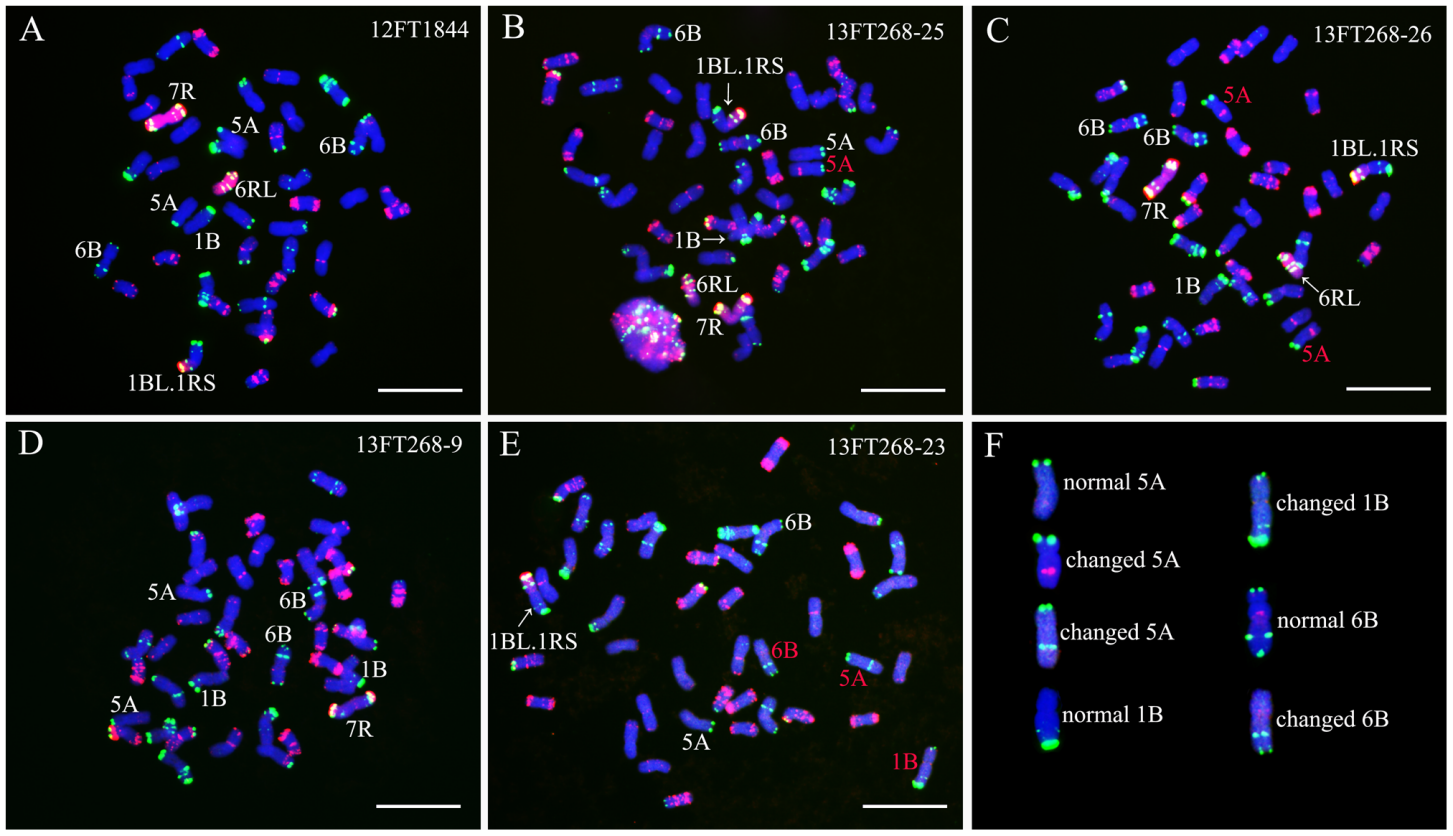
GISH and FISH using rye genomic DNA (red), Oligo-pSc119.2-1 (green) and Oligo-pTa535-1 (red) as probes on line 12FT1844 and its progeny. **A** GISH and FISH pattern of line 12FT1844. **B** GISH and FISH pattern of line 13FT268-25, representing lines with one changed 5A chromosome. **C** GISH and FISH pattern of line 13FT268-26, representing lines with two changed 5A chromosomes. **D** GISH and FISH pattern of line 13FT268-9, representing lines with normal 5A chromosomes. **E** GISH and FISH pattern of line 13FT268-23. **F** Cut-out chromosomes for the comparison between normal and changed wheat chromosomes. Changed wheat chromosomes are marked by red letters in B, C and E. Chromosomes were counterstained with DAPI (blue). Bars = 10 µm.

Line 12FT1844 contained a 1BL.1RS translocation chromosome, a 6RL arm and a 7R chromosome (Figure 6A). In line 12FT1884, it can be noted that both of the long arms of 5A chromosomes did not contain apparent intercalary Oligo-pTa535-1 and Oligo-pSc119.2-1 signals, the short arms of 1B chromosomes did not bear terminal Oligo-pSc119.2-1 signal and both of the 6B chromosomes bore obvious terminal Oligo-pSc119.2-1 sites (Figure 6A). Among the 30 randomly selected self-fertilized progeny of 12FT1844 (13FT268.1-13FT268.30), 13 lines contained one changed 5A chromosome whose long arm carried strong intercalary Oligo-pTa535-1 signal (Figure 6B, F), five lines contained two altered 5A chromosomes whose long arm carried strong intercalary Oligo-pTa535-1 signals (Figure 6C, F) and 11 lines contained normal 5A chromosomes (Figure 6D). Interestingly, in a line 13FT268-23, a 5A chromosome with obvious intercalary Oligo-pSc119.2-1 signal on long arm, a 1B chromosome carrying apparent terminal Oligo-pSc119.2-1 signal on short arm and a 6B chromosome without terminal Oligo-pSc119.2-1 signal on short arm were observed (Figure 6E–F).

**Figure 7:**
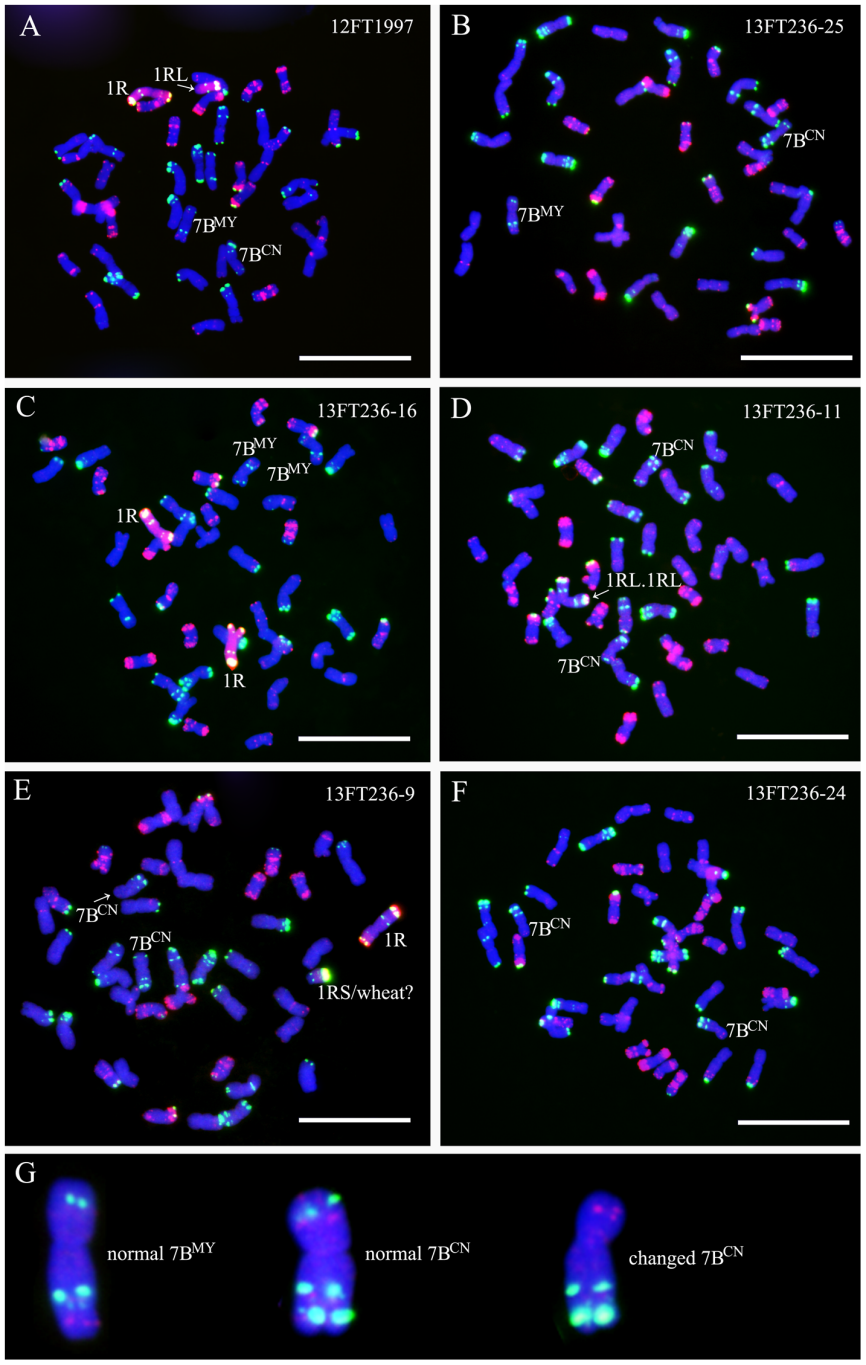
GISH and FISH using rye genomic DNA (red), Oligo-pSc119.2-1 (green) and Oligo-pTa535-1 (red) as probes on line 12FT1997 and its progeny. **A** GISH and FISH pattern of line 12FT1997. **B** GISH and FISH pattern of line 13FT236-25, representing lines with normal 7B^MY^ and 7B^CN^ chromosomes. **C** GISH and FISH pattern of line 13FT236-16, representing lines with two normal 7B^MY^ chromosomes. **D** GISH and FISH pattern of line 13FT236-11, representing lines with normal 7B^CN^ chromosomes. **E** GISH and FISH pattern of line 13FT236-9, representing lines with one changed 7B^CN^ chromosome. **F** GISH and FISH pattern of line 13FT236-24, representing lines with two changed 7B^CN^ chromosomes. **G** Cut-out chromosomes for the comparison between normal and changed 7B chromosomes. Changed wheat chromosomes are marked by red letters in E and F. Chromosomes were counterstained with DAPI (blue). Bars = 10 µm.

A 1R chromosome, a 1RL arm, a 7B^MY^ chromosome and a 7B^CN^ chromosome existed in line 12FT1997 (Figure 7A). Oligo-pSc119.2-1 signals could be observed on both of the short arms of 7B chromosomes (Figure 7A). Twenty-five seeds (13FT236.1-13FT236.25) were randomly selected from the self-fertilized progeny of 12FT1997 for FISH and GISH analyses. Among the 25 progeny, 15 lines contained a 7B^MY^ chromosome and a 7B^CN^ chromosome (Figure 7B), four lines contained two 7B^MY^ chromosomes (Figure 7C) and six lines contained two 7B^CN^ chromosomes (Figure 7D). Among the six lines, two lines carried one changed 7B^CN^ chromosome and two lines had two changed 7B^CN^ chromosomes. That is, the subtelomeric Oligo-pSc119.2-1 signals disappeared from the 7B^CN^S arm(s) (Figure 7E–G).

The data about the transmission frequency and structural variations of rye chromosomes were not shown because the focus of this study is on the structural alterations of wheat chromosomes.

## Discussion

### New type of wheat chromosomal structure variation

Recently, some new tandemly repeated sequences such as pTa-535 and pTa-713 were discovered [16]. pTa-535 is an especially valuable repetitive sequence for wheat chromosome identification [16]. Oligonucleotide Oligo-pTa535-1 was developed according to the nucleotide sequence of pTa-535 [15]. It has already been reported that Oligo-pTa535-1 combined with Oligo-pSc119.2-1 can identify all 21 wheat chromosome pairs and seven rye chromosome pairs because these two oligonucleotides can replace the roles of tandem repetitive sequences pTa-535 and pSc119.2, respectively [15]. In the present study, FISH analysis using probes Oligo-pTa535-1 and Oligo-pSc119.2-1 has revealed the structural variations of wheat 5A, 6A, 1B, 2B, 6B, 7B, 1D, 3D and 7D chromosomes in derivatives of wheat-rye hybrids. FISH analysis adopting pSc119.2 and pAs1 as probes has been used to investigate 22 wheat cultivars and a wheat line, and the results indicated that the hybridization patterns of chromosomes 4A, 5A, 1B, 2B, 3B, 5B, 6B, 7B, 1D, 2D, 3D and 4D were different [17]. Four hundred and sixty polyploidy wheat accessions were scored by C-banding and extensive chromosomal rearrangements were detected [18]. Although chromosomal rearrangements including translocation and inversion could be detected by C-banding technology [18], FISH analysis using repetitive sequences as probes is an effective method for detecting localized genomic changes [5]. 5AL arm with intercalary pSc119.2 or Oligo-pTa535-1 signal, 6BS and 3DS arms without terminal pSc119.2 signal, 1BS and 1DL arms with terminal pSc119.2 signals and centromeric T1B:2B chromosomes have already been reported [14]–[15], [17]–[18]. However, the 5AL arm carrying both strong intercalary Oligo-pTa535-1 and Oligo-pSc119.2-1 signals, 6AS arm with terminal Oligo-pSc119.2-1 signal, 7BS arm without subtelomeric Oligo-pSc119.2-1 signal and 7DL arm with intercalary Oligo-pSc119.2-1 signal have not been reported in common wheat (*T. aestivum* L.). Therefore, the changed 5A, 6A, 7B and 7D chromosomes in this study represent several new karyotype structures of common wheat chromosomes. The wheat lines containing new rearranged chromosomes are new materials for wheat breeding program.

### Wheat chromosomal alterations induced by rye chromosomes

Wide hybridization is one of the stresses that might trigger reorganization of the parental genomes [19]. Common wheat (*T. aestivum* L.) is formed through natural wide hybridization and allopoplyploidy. During the evolutionary process of common wheat, chromosomal structural variations such as 4AL-5AL-7BS, 2AS-4BS and 2AL-4BL translocations have been detected [20]–[21]. Wide hybridization between wheat (*T. aestivum*) and rye (*S. cereale*) is an important cytogenetic and breeding tool in wheat. The purpose of crossing wheat with rye is to enrich the cultivated gene pools of wheat. In fact, chromosomal rearrangement of wheat induced by wide hybridization is noteworthy. Chromosomal variations of wheat chromosomes induced by wheat-rye monosomic addition lines have been reported [14]. In this study, the wheat lines with changed wheat chromosomes also contained one or several rye chromosome(s). Both the previous study and this study indicate that rye chromosomes added to wheat background could induce modifications of wheat chromosomes. The question arises as to the possible cause of the structural variations of wheat chromosomes. The reciprocal translocation between 1B and 2B chromosomes might be caused by the 4R chromosome added into wheat genetic background, because wheat-rye monosomic addition lines can easily induce structural variation of chromosome and high frequency of chromosome translocation [22]. The other structural alterations of wheat chromosomes were revealed by FISH analysis using tandem repetitive DNA sequences as probes. It has been suggested that major structural chromosomal rearrangements including deletions, duplications, translocations and inversions are very often associated with cytogenetically detectable regions that are composed of repetitive DNA sequences [18]. Wide hybridization could accelerate repetitive DNA sequence evolution [23]. It has already been reported that retrotransposon-like sequences formed the junctions of tandem repetitive sequences [24]. It was supposed that the deletion and expansion of tandem repetitive sequences in wheat-rye addition and substitution lines might be related to retrotransposon [9]. Therefore, the structural variations of wheat chromosomes in this study might also be related to retrotransposon. Of course, further evidence for this hypothesis is needed. Localized rapid genomic changes involving loss or gain of pSc119.2 repeat in newly synthesized allotetraploid wheats have been reported and these localized systemic genomic changes may have played a role in karyotype stabilization [5]. Therefore, the biological function of repetitive DNA sequences in wide hybrids is worth studying. Undoubtedly, the structural modifications of wheat chromosomes in this study represent a new genetic variation of wheat genome. This kind of genetic variation might have the potential to impact positively on wheat improvement.

In conclusion, structural modifications of wheat chromosomes could occur in derivatives of wheat-rye hybrids. Perhaps, wheat lines with these new modified wheat chromosomes contain the potential value fro wheat breeding program. Additionally, the alterations of wheat chromosomes were displayed by the dynamic changes of tandem repetitive DNA sequences, therefore, these wheat lines are valuable materials for studying the biological function of tandem repetitive DNA sequences.

## Materials and Methods

### Plant materials

The octoploid triticale lines MK19-2 and MK25-2 were obtained by crosses between common wheat *T. aestivum* L. ‘Mianyang11’ (genomes AABBDD) and rye *S. cereale* L. ‘Kustro’ (genome RR).

A wheat-rye 4R monosomic addition line, 1-6-7B-2, was identified from the BC_2_F_3_ seeds that were derived from MK19-2×‘Mianyang11’. Some self-fertilized progeny of 1-6-7B-2 were obtained. Wheat lines 12FT1290, 12FT1378 and 12FT1844 were identified from the BC_1_F_3_ seeds that were derived from MK25-2×‘Mianyang11’. Wheat line 12FT1997 was identified from the BC_1_F_3_ seeds that were derived from MK25-2×‘Chuannong27’. ‘Chuannong27’ is a common wheat cultivar, which was released in Sichuan Province in 2007. The self-fertilized progeny of wheat lines 12FT1290, 12FT1378, 12FT1844 and 12FT1997 were also used and they were named 13FT294 13FT279, 13FT268 and 13FT236, respectively.

### Cytological techniques and *in situ* hybridization

FISH and GISH were used to analyze the mitotic metaphase cells of materials used in this study. The genomic DNA of rye ‘Kustro’, Oligo-pSc119.2-1 and Oligo-pTa535-1 [15] were used as probes. The genomic DNA of rye ‘Kustro’ was labeled with Texas Red-5-dUTP (Invitrogen). Oligo-pSc119.2-1 and Oligo-pTa535-1 were 5′ end-labelled with 6-carboxyfluorescein (6-FAM) and 6-carboxytetramethylrhodamine (Tamra), respectively [15]. Oligonucleotide probes were synthesized by Shanghai Invitrogen Biotechnology Co. Ltd. (Shanghai, China). The two synthesized probes were diluted by using 1×TE solution and the amount applied was operated according to Tang et al. [15]. The chromosome spreads of materials were prepared through the methods described by Han et al. [25]. Probe labeling and *in situ* hybridization were also operated according to Han et al. [25]. Images were taken using an epifluorescence microscope (BX51, Olympus) equipped with a cooled charge-coupled device camera operated with HCIMAGE Live software (version 2.0.1.5) and processed with photoshop CS 3.0.
